# In this Together: International Collaborations for Environmental and Human Health

**DOI:** 10.1017/jme.2023.82

**Published:** 2023

**Authors:** Jaime S. King, Joanna Manning, Alistair Woodward

**Affiliations:** 1: THE UNIVERSITY OF AUCKLAND, AUCKLAND, NEW ZEALAND

**Keywords:** Climate Change, Environmental and Human Health, Economic Case for Climate action, International Collaborations

## Abstract

Climate change exacts a devastating toll on health that is rarely incorporated into the economic calculus of climate action. By aligning health and environmental policy and collaborating across borders, governments and industries can develop powerful initiatives to promote both environmental and human health.

“Every nation in the world needs to work together to stabilize the Earth’s climate. We either all succeed together or all fail together.”

*—Joëlle Gergis* ^1^

COVID-19 taught us that the health of our bodies, our economies, and the planet are inextricably linked. As we face the bigger and more difficult challenge of climate change, this lesson should ring loudly in our ears and remind us that that now, more than ever, we must reach across borders and oceans to come together for the sake of the planet and the betterment, if not the survival, of all human beings.

The Intergovernmental Panel on Climate Change (IPCC) determined that annual global emissions must be reduced to fifty percent of 2010 levels by 2030 to limit temperature increases to well below 2^o^C with a target of 1.5^o^C.^2^ By signing the 2015 Paris Agreement, 220 nations agreed to establish national climate action plans that set long-term emissions goals, known as a nationally determined contributions (NDC), which will be updated in five year cycles.^3^

Despite these commitments, the world is not on track to limit warming to 2 degrees, let alone the more ambitious path to 1.5 degrees.^4^ Although the Paris Agreement requires signatories to establish NDC emissions pledges, it has not had sufficient international enforcement mechanisms to mitigate global warming or prevent states from failing to reach their commitments, both in terms of emissions targets and financial contributions.^5^ In 2021, global emissions, having reduced some 10 percent from 2019 levels in developed nations during the COVID-19 pandemic, rebounded by six percent, reaching an all-time high.^6^ In 2022-2023, many countries have placed their climate change goals on the back burner after being hit by an international energy crisis and a cost-of-living crisis, in the midst of attempting to recover from the pandemic. As a result, current mitigation efforts are inadequate to avert a temperature rise associated with poorly understood risks of very serious outcomes.^7^

In the meantime, the toll that climate change takes on human health and its associated financial costs has become increasingly apparent. The WHO estimates that 13 million people die annually as a result of modifiable environmental factors, many of which are climate-sensitive, amounting to nearly a quarter of all global deaths.^8^ The direct damage costs of climate change are projected by WHO to reach between US$2-4 billion per year by 2030.^9^ Unfortunately, every increment of warming above 1.5^o^C will amplify destructive effects on lives and health.^10^ As the Lancet Countdown on Health and Climate Change’s 2022 report observes, “the window of opportunity to limit temperature rise is rapidly closing. ” ^11^

But it has not closed yet. This article aims to bolster the argument that immediate and long-term harms to health arising from climate change should be included in decision-making regarding climate change mitigation. If world leaders were reluctant to pass legislation to preserve the planet due to the economic cost and inconvenience, perhaps they would do so to avoid the devastating health harms and the immense costs arising from them. Consistent with the theme of international collaboration, we advocate for cooperative and collaborative initiatives at all levels: global, international, domestic, and subnational. Our rationale is that broader and deeper collaboration at all levels may promote synergies, best practice bandwagons, and climate leaders to hopefully compound beneficial outcomes (environmental and human health). International collaboration to attack this ubiquitous threat recognizes that, as never before, we are all in this together.

But it has not closed yet. This article aims to bolster the argument that immediate and long-term harms to health arising from climate change should be included in decision-making regarding climate change mitigation.^12^ If world leaders were reluctant to pass legislation to preserve the planet due to the economic cost and inconvenience, perhaps they would do so to avoid the devastating health harms and the immense costs arising from them. Consistent with the theme of international collaboration, we advocate for cooperative and collaborative initiatives at all levels: global, international, domestic, and subnational. Our rationale is that broader and deeper collaboration at all levels may promote synergies, best practice bandwagons, and climate leaders to hopefully compound beneficial outcomes (environmental and human health). International collaboration to attack this ubiquitous threat recognizes that, as never before, we are all in this together.

This article proceeds in four parts. Part I provides the evidence base for the relationship between the environment, climate change and health. Part II analyzes the economic costs of the health impacts of climate change. In response, Part III argues in favor of broad international collaboration to address the dual public health and climate change crises. Finally, Part IV offers examples and recommendations for international collaboration to promote human and environmental health.

## I. The Relationship between the Environment, Climate Change, and Health

Described by the Director-General of Health of the WHO as “the single biggest health threat facing humanity,”^13^ overwhelming evidence now demonstrates the profound deleterious health impacts arising from climate change. The Lancet Countdown on Health and Climate Change has published reports for seven years, monitoring the associations between health and climate change. A key finding of its 2022 report is that: ”climate change is undermining every dimension of global health monitored, increasing the fragility of the global systems that health depends on, and increasing the vulnerability of populations to the coexisting geopolitical, energy, and cost-of-living crises.”^14^

Climate change has both direct and indirect relationships with health harms. Studies have increasingly established the direct effect of anthropogenic climate change on health through exposure to increasingly frequent and extreme weather events,^15^ such as floods,^16^ heat waves,^17^ droughts,^18^ and severe wildfires.^19^ In some locations, temperature changes are expanding mosquito and other disease-vector habitats and lengthening the transmission season of arthropod-borne and water-borne infectious diseases, placing populations at higher or new risk of infection.^20^ Climate scientists are now able to conclude with confidence that human-generated global warming increased the likelihood of and exacerbated many of the extreme weather events in 2021 and 2022 causing death and devastation across every continent.^21^ Taking health-related mortality as an example, a 2021 landmark attribution study of 43 countries estimated that more than one third (37 percent) of heat-related deaths during warm months in the period 1991-2018 were attributable to human-induced climate change, and that increased mortality was evident on every continent.^22^

Climate change also contributes to ill health through worsening air pollution, which is now recognized to be among the leading risk factors for premature mortality.^23^ Poor air quality kills over seven million people annually and is the second leading cause of death from non-communicable disease globally.^24^ In 2018, it is estimated that over 90 percent of people living in cities worldwide were breathing air containing levels of air pollutants that exceeded WHO’s guidelines.^25^ Air pollution is predicted to increase in the coming years due to continued fossil fuel emissions and exposure to smoke and dust caused by longer and more intense droughts and more frequent wildfires.^26^

In addition to directly harming health, climate change, as a result of economic disruption and population displacement, also undermines many of the factors necessary for good health outcomes, such as reliable incomes, decent housing, connection to place, and access to functional social services.^27^ These and other so-called social determinants of health may be jeopardized by extreme weather and long-term climate shifts.^28^ A distinctive feature of climate change is that its causes are largely outside the control of the community in which an individual lives, but largely result from policies and practices of governments and businesses in other parts of the world. In addition to acute events such as floods, fires, and heat-waves, these are slower onset threats, such as air pollution, changed rainfall patterns, water and soil degradation, land and marine ecosystems changes, and shifts in the distribution of temperatures that increase the transmission of infectious diseases.^29^ In some places, warmer temperatures shorten crop growth seasons, contributing to the decline in global yields for major staple crops, and threatening food security from marine sources.^30^ Food insecurity affected 720-811 million in 2020, with 98 million more people reporting moderate to severe food insecurity.^31^ Malnutrition is predicted to be one of the greatest threats to human health resulting from climate change, exacerbating existing health inequities between and within populations.^32^

The Marmot reports of 2010 and 2020 documented how the social and environmental determinants of health are largely responsible for disparities in health outcomes and inequities between individuals and population groups.^33^ Unequal access to appropriate health care is important of course, but what happens within the health system accounts for a minority (about twenty percent, on one estimate) of differences in health status, and factors outside the health system exert a much stronger influence.^34^

While most research has focused on the multiple connections between climate change and physical health, there is mounting evidence of its direct and indirect impacts on mental health and psychosocial wellbeing.^35^ The strongest evidence demonstrating its impact on mental health is in the context of emergency and disaster management, especially extreme weather events.^36^ Disasters can increase stress, strain social relationships, and introduce or worsen existing mental health conditions, such as anxiety, depression, and alcohol and substance abuse.^37^ These conditions are often compounded by prolonged breakdowns in societal institutions, sanitation and food distribution systems, critical public health infrastructure, and health service delivery, all of which can further impair mental health.^38^ In addition, many health systems are poorly resourced, with mental health support and treatment often particularly inadequate.

Climate change also aggravates many social and environmental risk factors for mental health and psychosocial wellbeing, such as housing vulnerability, air quality, food and water security, and income loss.^39^ The reciprocal relationship between poverty and mental health is well-established in the literature.^40^ Being poor and living in a society with high wealth inequality can create mental health problems, and mental health problems can also result in people falling into poverty.^41^

Unfortunately, climate change amplifies health and economic inequities on a global scale. Just as Covid-19 disproportionately affected the most vulnerable, the health impacts of climate change are also inequitably distributed within societies and on a global scale.^42^ The most at-risk populations include economically disadvantaged groups, some communities of color, immigrant groups, Indigenous peoples, children and pregnant women, older adults, outdoor workers, and persons with disabilities and pre-existing medical conditions.^43^ Peoples in developing countries suffer a disproportionate burden of the adverse impacts of climate change, despite these countries historically emitting low levels of greenhouse gases, because of heightened physiological susceptibilities, greater exposures, and the fewest resources to prepare for or protect themselves from its effects.^44^ The Lancet Countdown’s 2021 report found, ”The concurrent and interconnecting risks posed by extreme weather events, infectious disease transmission, and food, water, and financial insecurity are over-burdening the most vulnerable populations.”^45^

To sum up, climate change is already adding to the burden of ill-health world-wide, and impacts are projected to increase steeply in the future. Conservatively, the WHO estimated that climate change would result in an additional 250,000 deaths each year between 2030 and 2050 from child malnutrition, malaria, diarrhoea and heat stress, and the health of hundreds of millions more people would be affected.^46^ Furthermore, approximately 100 million people could be pushed into poverty every year due to the health impacts of climate change, with major impacts on mortality and morbidity.^47^ In response, states must take urgent, robust action to limit climate change in ways that simultaneously promote and protect human health, with primary responsibility falling on wealthy nations.^48^ While this conclusion is well-supported elsewhere in the literature on scientific, moral, and ethical grounds,^49^ Part II explores the economic argument in favor of health-focused climate mitigation.

## II. The Economics of Climate Change and Health

Economic cost is the most frequently touted reason for failing to take the necessary steps to mitigate climate change.^50^ In fact, the cost of climate mitigation initiatives is so readily assumed to outweigh the benefits to living individuals, that many climate advocates have started to focus the argument on the impact on children and future generations.^51^ Yet failing to mitigate climate change presently generates substantial economic harms through increased healthcare costs, loss of labor and productivity, and other damage caused by climate-related health events that are poorly accounted for.^52^ A 2021 joint report by The Medical Society Consortium on Climate and Health, National Resources Defense Council, and the Wisconsin Health Professionals for Climate Action concluded that in the United States alone the “health costs of air pollution and climate change far exceed US$800 billion per year and are expected to become even more expensive in years to come without a stronger societal response to address this crisis.”^53^ The Lancet Countdown on Climate Change estimated that extreme weather events alone caused damage worth US$253 billion in 2021, with 84 percent of these losses occurring in very high-human development index (HDI) countries, but with the economic burden disproportionately impacting low HDI countries where almost all losses are uninsured.^54^ Further, a report issued by United in Science calculated that climate-related disasters currently result in an estimated US$200 million in economic losses a day.^55^ It has been estimated, for instance, that boosting active transport in the UK, in addition to cutting greenhouse emissions, would save the National Health Service £17 billion over 20 years.^56^ While debates over climate policy should undoubtedly consider and incorporate harms to future persons, much of the cost of climate mitigation can be justified in more direct and current economic impacts.

Unfortunately, the economic costs arising from climate-related harms are infrequently incorporated into climate policy. For instance, the social cost of carbon (SCC) is “arguably the single most important concept in the economics of climate change,” yet the full cost of the health impacts of climate change are not currently accounted for in the SCC.^57^ According to economic theory, the SCC represents the marginal social damage caused by emitting one metric ton of carbon dioxide, and therefore it is the price that should be put on carbon dioxide emissions to reduce them to socially optimal levels.^58^ The SCC is commonly used in integrated assessment models that analyze the state of scientific evidence to inform climate policy. Notably the SCC has not accounted for the cost of climate change’s impact on human mortality. By incorporating the human mortality costs into economic projections of climate change and using them to recalculate the SCC, researchers at Columbia University determined that the 2020 SCC increased seven-fold from $39 to $258 USD per metric ton of carbon emitted, which shifted the economic optimal climate policy to full decarbonization by 2050.^59^

Likewise, while the health benefits of mitigating climate change are well-established, the economic impact of achieving those benefits has, until recently, been largely absent from policy debates regarding the economic implications of climate change initiatives.^60^ A 2018 ground-breaking study by Anil Markandya and colleagues found that at a global level the health “co-benefits”^61^ of implementing several air pollution mitigation scenarios substantially outweighed the cost of the mitigation.^62^ While the current NDC commitments were insufficient to meet the Paris Agreement targets, the study estimated that the total cost of reducing emissions to achieve the 2^o^C target ranged from US$22.1 trillion - US$41.6 trillion (0.5-1 percent of global GDP) and the cost of achieving the 1.5^o^C target ranged from US$39.7 trillion - US$56.1 trillion (1-1.3 percent global GDP)^63^ On a global level, the health co-benefits of achieving the required emissions reductions in each scenario far exceeded the cost of achieving the reductions, and in one scenario the health co-benefits were more than double the costs.^64^ The mitigation costs and health co-benefits, however, were not evenly distributed between the five regions studied, which included China, Europe, India, the United States and the rest of the world (ROW). In fact, for the US, the ROW, and in many instances Europe, the health co-benefits alone would not entirely outweigh the mitigation costs required to hit the targets.^65^ This should not dissuade those nations from action, however, as the health co-benefits remain significant in the broader fight against the existential crisis that is climate change.

Economic models calculating cost-benefit ratios of alternative climate policies are limited instruments, since much of value (including good health and a full life) is difficult to cost, but at least such models should acknowledge the damage to health caused by failing to meet emissions targets, as well as the co-benefits for population health of meeting them.^66^ Given that recent studies estimate that stabilizing emissions would require only a modest reduction (approximately 0.5-1.5 percent) of world production,^67^ these costs should pale in comparison to the possible catastrophic impacts to human and environmental health arising from unmitigated climate change.^68^ In Part III, we argue that concerted climate initiatives at all levels, subnational, bi- and multilateral, will be required — and are in fact emerging — to meet this unprecedented threat to human health.

## III. International Collaboration: The Need for a Global and Multilevel Response

Responses to the climate crisis to date, in terms of mitigation and adaptation, have largely been at the state (national) level: setting domestic targets, establishing in-country emissions schemes, completing national assessments, and developing national health and climate change adaptation plans. Internationally, global bodies have supported and pressured countries to do more within their borders in terms of setting and achieving more aggressive targets, committing sufficient “loss and damage” funds to assist developing nations stricken by climate impacts,^69^ and adapting to the effects of climate change. While these efforts at the state level have brought significant (but insufficient) gains in recent years, this approach is limited by the inability of international law to require states to enter international agreements or comply with ultimately voluntary targets.^70^ Broader international collaboration is required to mitigate, prepare, adapt, and address the impacts of climate change.

The need for international collaboration is especially apparent with respect to the environmental determinants of health. The traditional wisdom has been that national governments are best positioned to tackle the social determinants of health through socially progressive domestic policies, such as a “Health in All Policies” approach.^71^ There are global aspects to other social determinants of health (such as educational attainment and homelessness), but both the causes of climate change and its consequences are outside the control of any one nation. Air pollution, extreme weather, and sea level rise are ubiquitous and do not halt at national borders. In many instances, the nations that are most vulnerable to the impacts of climate change contributed very little to its development.^72^ No nation, no matter how populous, powerful or wealthy, has the ability to address the health effects of climate change on its own.^73^ While it is clear that nations must come together to share resources and information, collaborate on innovative technology, and mutually bind themselves to their commitments, just how that will happen remains elusive, but is starting to come into view.

Recent scholarship advocates the advantages of broad, dynamic, multilevel approaches to climate action, beyond traditional top-down regulation directed by international institutions at national governments.^74^ Such approaches can operate across national borders, both internationally and subnationally, facilitating information distribution, efficient funding and resource allocation, and binding international agreements with climate-related terms.” Best practice” recommendations emphasize collaborative responses that break down disciplinary silos, such as the environment and public health, to address a threat as ubiquitous, intractable, and inimical to human life and wellbeing as climate change.^75^ International collaboration represents an area of largely untapped potential which could help generate momentum and realize synergies for real progress globally on climate change and health.

## IV. Opportunities for International Collaboration on Climate Action and Health

International and subnational collaboration to promote human and environmental health can take numerous forms and levels of commitment. This part analyzes a range of collaborative policies and practices that progress from lower-stakes initiatives such as information sharing to international and subnational agreements to binding international trade agreements to green taxes and tariffs.

### A Information Sharing

Efforts to mitigate the health effects of climate change should not occur in a vacuum. Given the necessity of rapid changes in manufacturing, transportation, human consumption patterns, and waste production and disposal, governments and industry members must increase their levels of cooperation and information sharing about the health harms associated with climate change and their costs, methods to green the healthcare industry, litigation strategies to enforce the right to environmental health, and model policies and practices.

#### 1. THE HEALTH HARMS OF CLIMATE CHANGE AND THEIR COSTS

Sharing research and information on the health harms associated with environmental degradation and climate change, as well as their societal costs, is essential for nations to build the case for immediate action to protect the environment. As discussed, the health-related costs arising from climate change and the health co-benefits of robust climate action are often omitted from cost-effectiveness analyses of climate change interventions.^76^ International alliances to fund, monitor, and disseminate research on the impact of climate change on health and its costs can help convince policymakers and the public of the economic case for rapid mitigation of climate change. Drawing on an international collaboration of 250 multidisciplinary researchers from approximately one hundred academic institutions and UN agencies worldwide, The Lancet’s Countdown series sets the gold standard by tracking progress on health and climate change across five domains and an ever-expanding range of indicators (43 in 2022), setting the gold standard.^77^ Furthermore, such health-based alliances may encourage nations to recommit to their emissions targets under the Paris Agreement and their funding contributions to developing nations to help them cut emissions and adapt to changing conditions.^78^

The argument for health-based environmental mitigation should have the greatest effect in nations with public health systems, as climate change serves as a “health risk multiplier” for the chronically ill and other vulnerable populations, increasing costs and widening existing gaps in health and economic equity.^79^ Public health systems may falter under both increased health services utilization and the increased costs arising from climate-related health conditions. The case is also compelling, however, in states with large private health systems. In the U.S., the broader economy already struggles under the weight of healthcare costs, which stagnate wages, diminish corporate revenues, reduce spending and investment in other areas of the economy, and require ever greater percentages of state and federal budgets.^80^ With healthcare expenditures accounting for nearly 18 percent of GDP in 2019, the U.S. must find ways to reduce healthcare spending.^81^ Reducing healthcare spending in ways that align with the U.S.’s commitments under the Paris Agreement and the climate change mitigation incentives included in the Inflation Reduction Act can shift the economic calculus for businesses and government entities. Overall, nations should ensure that the measures of climate impact used in policy fully incorporate an increasingly comprehensive set of international research on the health harms arising from climate change.

Beyond sharing research for use in economic cost models, nations should collaborate to translate and disseminate research on the health impacts of climate change to public health professionals and other medical practitioners to help them prepare health systems and treat patients. The UN currently offers a handful of short courses and seminars on the health impacts of climate change, such as *Climate Change Negotiations and Health* and *Human Health and Climate Change*, which can help broadly disseminate information.^82^ To promote better cooperation and collaboration between climate scientists and health professionals, as well as provide valuable resources to policymakers, journalists, academics, and the public, the WHO and the World Meteorological Organization (WMO) jointly launched ClimaHealth.info, the first global platform dedicated to the climate and health, in October 2022.^83^ The recently launched website provides an essential platform for international collaboration on climate and health that can significantly expand knowledge of health impacts, treatment and adaptation approaches, and policy tools.^84^

#### 2. METHODS TO GREEN THE HEALTHCARE INDUSTRY

Healthcare providers and government regulators should also share information regarding ways to reduce emissions and other environmental harms arising from the healthcare industry itself. Despite its essential mission to promote and protect health, health care is paradoxically getting dirtier through ever-increasing resource use, waste, and emissions.^85^ For example, the U.S. health sector contributes an estimated 8.5 percent of U.S. greenhouse gas emissions (a larger percentage than that of any other country)^86^ and global health sector emissions contribute approximately 5.2 percent of global emissions.^87^ Pollution from healthcare energy use in the U.S. results in the loss of an estimated loss of 405,000 disability-adjusted life-years annually, similar to the disease burden from preventable medical errors.^88^ Importantly, these emissions are not strongly correlated with higher quality of care, making significant reductions feasible without compromising patient care.^89^

At the COP26 Health Programme, 45 countries, including the U.S., committed to “greening” their health systems by transitioning to low carbon or net zero carbon health systems.^90^ To achieve this, health policymakers and managers need to analyze the public health infrastructure and health system interventions for ways to decarbonize health care, such as through increasing energy efficiency of health care facilities, improving waste management practices, using renewable energy, and reducing emissions throughout the supply chain.^91^

Minimizing the environmental impact of healthcare activities requires innovation, rethinking and redesigning all aspects of health care delivery. National governments can leverage their unique positions as health care service providers, purchasers, regulators, and sponsors of research, education, and training to drive innovation and imminently reduce the environmental harms arising from the healthcare industry.^92^ Subnational entities can also collaborate with one another to drive change. For instance, the UN Race to Zero, a UN-backed initiative rallied non-state actors including companies, cities, regions, investors, and educational institutions to take immediate action to reduce carbon emissions and create a zero-carbon world by 2050.^93^ The initiative has been joined by 65 healthcare institutions representing over 3,200 healthcare facilities across 18 countries and over a quarter of major pharmaceutical companies and medical technology companies.^94^ The healthcare industry has great potential to lead other industries in the transition to a carbon-zero world, as it has been doing for some time.

#### 3. LITIGATION STRATEGIES

Like industry-focused initiatives, climate change litigation has gained significant momentum in recent years, both through a near doubling in the number of cases brought and through successful litigation strategies.^95^ Cases such as *Billy et al. vs. Australia*, *Stitching Urgenda vs. The State of the Netherlands*, *Future Generations v. Ministry of Environment Colombia,* and *Milieudefensie et al. v. Royal Dutch Shell* have held nations and private actors accountable for failing to meet their climate change obligations.^96^ In July 2022, the UN General Assembly adopted resolution 76/300 affirming the human right to a clean, healthy, and sustainable environment.^97^ Certification of the right promises to accelerate lawsuits against governments and major polluters.^98^ Over one hundred cases have been brought against national governments for human rights violations arising from failures to mitigate climate change, with thirty asserting a right to a healthy environment.^99^

These cases provide litigation strategies that use harm to human health as a mechanism to address local environmental violations as well as broader failings by government and industry to mitigate climate change. For instance, on November 25, 2022, over six hundred people born between 1996 and 2015 filed a class action against Sweden in the European Court of Human Rights, arguing that Sweden’s failures to meet its obligations to reduce greenhouse gas emissions violated their rights to life, private and family life, non-discrimination, and property under the European Convention of Human Rights.^100^ The plaintiffs specifically alleged human rights violations from the significant impacts on human health arising from longer and more intense heat waves, greater precipitation and flooding, and longer tick and mosquito seasons arising from shorter and warmer winters.^101^ If successful, plaintiffs could bring similar claims for violations to health arising from nations failing to meet their greenhouse gas emissions obligations across Europe. Likewise, fifty representatives of the village of Kaboedin Thailand, members of the indigenous Karen tribe, filed a lawsuit against the Expert Committee on Environmental Impact Assessments (EIA) and the Office of Natural Resources and Environmental Policy and Planning claiming the falsification of an EIA report in 2020 which led to the continued approval of a coal mining project in the village and the violation of the right to a healthy environment.^102^ On September 23, 2022, the Administrative Court temporarily suspended all coal mining activities in Kaboedin pending final judgment in the case, and also reaffirmed the right to live in a good environment in line with the right to a healthy environment.^103^ These cases, and numerous others like them, can make dramatic headway not just in an individual country, but globally.

Historically, international cases of this type would be challenging to access in a timely manner, especially for indigenous populations, youth, and those without ready access to foreign lawyers and an interpreter. However, climate organizations, like Climate Change Litigation Databases and Climate Change Laws of the World, have begun to aggregate, categorize, and analyze the dramatically increasing number of climate cases filed in domestic courts around the world, providing real-time access to court documents and case summaries in English.^104^ International databases that aggregate climate change cases and court documents could provide similar summaries and court document translations in a variety of languages to promote successful climate litigation strategies, connect plaintiffs, and share resources to further climate litigation worldwide.

#### 4. MODEL POLICIES AND PRACTICES

Likewise, governments, climate and health advocacy organizations, and corporations striving to improve sustainability should share best practices and model policies to achieve the dual health and environmental benefits arising from well-chosen climate actions. Here we provide examples of active transport policies at the nexus of health and climate change.

Transportation is a prime target for policy reform for two reasons. The proportion of greenhouse gas emissions generated by the transport sector is substantial (about 17% globally and growing more quickly than any other sector), and the means to reduce emissions from transport are well-known and can be implemented at scale, rapidly. They have been described under the headings of Avoid, Shift and Improve.^105^ “Avoid” refers to environmental and economic changes that make vehicle travel unnecessary, such as increasing urban densities and work schedule flexibility to reduce commuting. “Improve” describes technologies that reduce the carbon-density of vehicle travel, such as electric cars and improved engine efficiency. Finally, “Shift” substitutes low-emission modes of travel (e.g. walking, cycling, and public transport) for high-emission travel (e.g. gas powered motor vehicles).

Interventions to increase active transport, such as walking and cycling, illustrate well the synergies between emissions reduction and health promotion.^106^ For example, Mueller and colleagues found that expanding the cycling networks in 167 European cities so that cycling accounted for approximately 25 percent of all trips taken (amounting to about 315 km/100,000 persons) would reduce premature mortality by over 10,000 deaths a year, due mostly to lower rates of mortality from cardiovascular causes.^107^ Active transport is also associated with mental health benefits. Active commuters tend to be more satisfied with their trips than those who travel to work by other modes — indeed cyclists are frequently reported to be the happiest commuters of all.^108^

Transitioning to more active transport is feasible, especially in urban environments where many trips cover short distances. In New Zealand, in 2003-2006, 29 percent of trips by adults aged 18-64 years by car in urban areas were 2 km or less, and 70 percent were 7 km or less. If just 5 percent of short car trips (< 7 km) were moved to bicycle, researchers estimated this would save 116 premature deaths and avoid burning about 22 million liters of petrol and diesel annually.^109^ In terms of emissions reduction, regular addition of just one trip a day by bike reduced lifecycle transport emissions by 14 percent in a recent European study. The same European study found that the transport footprint of “cyclists” was 80 percent less than that of “non-cyclists.”^110^

Given the dual benefits of active transport, identifying potential interventions, evaluating their effectiveness, and disseminating evidence-based model policies internationally can facilitate rapid implementation. A 2019 review aggregated and categorized strategies to encourage active transportation and reduce pollution, evaluated evidence of their effectiveness, and proposed several policy options for implementation.^111^ Interventions included car free cities and days, urban design improvements, green spaces, and investments in public transport.^112^ The strongest evidence supported separating cyclists and pedestrians from motor vehicles on paths that are wide, smooth, connected and lead to popular destinations.^113^

The effectiveness of mode shift interventions increases if there are “sticks” (deterrents to use of motor vehicles) as well as “carrots” (such as favorable street changes). Policies that restrict or discourage motor vehicle use, such as low emission zones and congestion charging, which introduce financial penalties for driving a car in certain areas at certain times, can promote active transport. The rapid growth in the number and quality of electric bicycles has boosted the number of people who ride (more women, more older people), and increased the range, variety, and frequency of e-bike trips.^114^ International organizations should create databases of mitigation and adaptation measures of this kind that aggregate research on the cost-effectiveness of different policies and practices, implementation guidance, and model regulations and legislation, and translate them into a variety of languages.

#### 5. INTERNATIONAL AGREEMENTS

Another form of collaboration ripe for significant expansion are bi-lateral and multi-lateral agreements to provide mutual assistance and resources in the event of natural disasters. For example, the USDA Forest Service and Australia have a reciprocal agreement to exchange fire management resources, including deploying firefighters and providing technical and operational support, to assist with each other’s wildfire suppression efforts during their respective wildfire seasons.^115^ In July 2021, an Australian Boeing 737 airtanker with personnel and equipment arrived in California to fight fires during the devastating 2021 season. It followed recent deployments of several hundred U.S. federal wildland firefighters and fire managers to Australia from December 2019 through the spring of 2020, to assist with Australian wildfires.^116^ These international agreements often result from long-standing working relationships based on a history of co-operation.^117^ So that the benefits can be expanded and shared more widely, hopefully these first steps will lead to a broadening of such cooperative activity into other areas, such as exchange agreements for emergency medical supplies and personnel, and to include multiple international partners, especially from low- and middle-income countries.

Cooperation can extend beyond disaster management and adaptation, into collaborative efforts to help states work together towards achieving their mitigation goals. For example, the Governor of California, Gavin Newson, has been active in establishing new international climate partnerships with multiple countries, including New Zealand, China, and Japan, to advance global climate leadership.^118^ He committed California to several agreements and memoranda of cooperation with the aim of advancing climate and clean energy priorities and strengthening trade relations.

In May 2022, Governor Newsom and New Zealand’s Prime Minister Ardern signed a non-binding Memorandum of Cooperation that aims to reduce pollution, bolster the clean economy, accelerate the transition to clean energy and zero-emission vehicles, and promote nature-based solutions, while emphasizing community resilience and partnership with indigenous leaders.^119^ Both jurisdictions share a common objective to achieve carbon-neutrality by 2050. The memorandum specifies areas of focus for cooperation, including transport electrification, emissions trading schemes, climate-smart agriculture, clean energy, nature-based solutions, and identifies specific activities and initiatives for co-operation, such as sharing knowledge, expertise and best practices, undertaking joint research and policy design, and collaborating on particular projects that help meet each other’s targets.^120^ While a voluntary initiative at present, the parties regard the Memorandum as establishing a stronger framework for economic cooperation and trade.^121^ Such agreements are a good first step which could pave the way for a new kind of binding, “environmentally-conditioned” trade agreement, in which governments commit to condition their bilateral trade practices on environmental protection and meeting their international obligations. A paradigm example is the EU-NZ free trade agreement, considered next.

#### 6. ENVIRONMENTALLY-CONDITIONED TRADE AGREEMENTS

The EU and New Zealand introduced a first-of-its-kind environmentally-conditioned free trade agreement (the EU-NZ Agreement) in 2022.^122^ This type of free trade agreement provides a mechanism to bind nations’ environmental actions to their trade and economic well-being and to create incentives for international collaboration on climate mitigation efforts and beyond. The EU-NZ Agreement reflects New Zealand’s recently adopted “Trade for All” policy statement, which aims to align trade practices with New Zealand’s values related to the environment and climate change, partnership with Māori, health, education, intellectual property, and labor rights.^123^ In Chapter 20 of the EU-NZ Agreement, the Parties acknowledge the commitments repeatedly made to sustainability by both New Zealand and the EU through a variety of international agreements and resolutions, including the Rio Declaration on Environment and Development, the Johannesburg Plan of Implementation of the World Summit on Sustainable Development of 2002, and the Paris Agreement.^124^ The Parties also recognize that “sustainable development encompasses economic development, social development, and economic protection, all three being inter-dependent and mutually reinforcing,”^125^ and emphasize the need to “enhance the mutual supportiveness between trade and environmental policies.”^126^ To that end, the EU-NZ Agreement establishes that each Party will: 1) establish laws and policies that meet their current environmental commitments; 2) provide high levels of environmental and labor protections and continuously strive to improve those levels; 3) not weaken or waive its current protections to enhance trade or investment; and 4) not fail to enforce its environmental and labor laws in ways that could affect trade or investment.^127^ The EU-NZ Agreement imposes these requirements and more specific conditions on the trade-related aspects of climate change mitigation and adaptation, fossil fuel subsidies, biological diversity, forestry, and fisheries and aquaculture.^128^ Specifically, the EU-NZ Agreement not only requires the Parties to take action within their own countries, but several provisions require them to reach beyond their borders to encourage further bilateral, regional and international collaboration to promote climate change mitigation, sustainable production and consumption, reduction of fossil fuel subsidies, and other environmentally friendly laws and policies.^129^

Environmentally-conditioned trade agreements add significant financial incentives for nations to invest in environmental protection and can create powerful trade alliances that may inspire other nations to meet their environmental commitments. Ideally, states could use trade agreements to provide preferential status to nations that enacted laws and policies that promote both environmental and human health, and also require collaboration on the development of pollution standards that appropriately account for health harms.

In the next section, we discuss the additional power of green tariffs and taxes, again pioneered by the EU, to incentivize environmental protection and climate action.

#### 7. TAXES AND TARIFFS

One of the powerful barriers to implementing climate mitigation efforts has historically been the cost. As a result, nations have begun creating financial incentives for each other to green their industries and meet their obligations under the Paris Agreement and other international agreements.^130^ Unlike international agreements, such as the Paris Agreement, which are often voluntary and even if not, provide minimal enforceability between nations, the imposition of green tariffs on goods entering the nation can provide additional financial pressure on states to protect the environment. The EU has led the way in using international trade mechanisms to advance climate mitigation.^131^

In December 2022, the EU became the first large economy to pass a “green” tariff on imports, which imposes a carbon tax on all imports that produce carbon emissions.^132^ The EU’s carbon border adjustment mechanism (CBAM) will initially target iron and steel, cement, fertilizers, aluminum, electricity, hydrogen, and other chemicals. According to Jozef Sikela, Minister of Industry and Trade of the Czech Republic who led the negotiations in the EU parliament, the CBAM promotes the import and sale of non-EU goods that meet the EU’s high climate standards and is a “key part of our climate action.”^133^ The CBAM also aims to reduce carbon leakage from EU companies avoiding the EU carbon emissions standards and existing carbon tax by moving high-carbon activities out of the EU and then importing those goods back into the EU.^134^ The overarching goal is to level the playing field with respect to trade and carbon emissions for goods produced within the EU and other nations.

While the CBAM represents a major step forward in terms of the European nations aligning to promote climate mitigation within and beyond the EU, the tariff has some limitations. First, it is limited to only a few industries, including cement, iron and steel, aluminium, fertilizers, electricity and hydrogen.^135^ Yet, Pascal Canfin, chair of the EU Parliament’s Environment Committee, noted that Parliament has plans to expand the CBAM to cover numerous other products, including processed products, like cars.^136^ Second, the CBAM will enter into its transitional phase in October 2023 which will include monitoring, reporting, and verification obligations, but full implementation with the pricing mechanism will not launch until 2026. In the meantime, many details remain uncertain.^137^ Until these are determined and the CBAM comes into force, the full implications of the tariff will be unknown. Finally, the CBAM does not eliminate the free allocation permits to produce carbon dioxide under the EU’s existing carbon trading scheme, but will instead phase them out between 2026 and 2034.^138^ The EU has granted free allowances to certain companies and industries, including those related to manufacturing, energy, and aviation, which limit their costs within the EU Emissions Trading System (ETS).^139^ In late December 2022, however, the EU Parliament and the European Council agreed to significantly increase reduction targets in emissions and free allocations, including steep declines in free allocations to the heavily protected aviation industry, eliminating them by 2026, and a more gradual decline in industry free allocations between 2026 and 2034.^140^ Researchers have estimated that CBAM implementation will increase the competitiveness of EU products and decrease that of non-EU products, but only if EU producers can absorb the excess demand.^141^ Further, there is speculation that the CBAM on its own may have little impact on global carbon emissions.^142^

Despite these limitations and uncertainties, Europe’s ‘green’ tariffs offer a strong model for how governments can work together to promote regional and global climate action. Governments seeking to extend the notion of green tariffs could adopt a broader version of the “polluter pays” principle^143^ that would incorporate the full scope of harms associated with greenhouse gas emissions, including the healthcare costs and economic losses arising from pollution-induced harms to health. By extending the CBAM to require similar tariffs on imports in non-European nations, nations could set a broader global standard for manufacturing and have a greater impact on global carbon emissions. Expanding both the use and the scope of green tariff schemes to incorporate health impacts has the potential to further shift the economic incentives associated with climate mitigation at a global level.

We urge the case for international cooperation and collaboration as an underdeveloped direction for policy and action, in addition to aspirational state compliance with voluntary targets. The first green shoots are appearing, from relatively low-stakes activities like information-sharing of best practices through to exciting developments which could really “move the needle,” such as binding international trade agreements to comply with climate goals and strong financial incentives like green tariffs. Private markets can also play a part if investors take a medium to long term view on financial investments and sustainability.

#### 8. INTERNATIONAL BUSINESS AND INVESTOR COLLABORATION

Beyond governmental actors, businesses and investors can also share resources, best practices, and lobbying strategies to promote climate action both domestically and internationally. After decades of failing to use their corporate influence to advocate for policies to address climate change and promote sustainability, many businesses have changed course after witnessing the extreme climate-related events in the last several years and the financial havoc they reeked. A 2022 report that examined the climate-related risk management, governance, and lobbying practices of S&P 100 companies found that in the last three years, half of the 104 companies analyzed advocated for climate policies that align with the Paris Agreement.^144^ The report also found, however, that very few of those companies publicly supported the Inflation Reduction Act of 2022, the most significant climate legislation in U.S. history, and far fewer publicly addressed the role that their trade organizations have played in obstructing climate action.^145^ International collaboration between investors, industry, and governments can help promote behavior changes within industry worldwide, but such commitments must be made in earnest.

Global networks of investors have begun to collaborate to place additional pressure on some of the world’s largest industrial emitters of greenhouse gases to force them to reduce their greenhouse gas emissions to sustainable levels.^146^ For instance, Climate Action 100+ is “an investor-led initiative to ensure the world’s largest corporate greenhouse gas emitters take necessary action on climate change.”^147^ The collaboration of 700 international investors aim to ensure that the companies they invest in and own help achieve the goals of the Paris Agreement and accelerate the transition to net-zero emissions by 2050 by making it a critical part of their business strategy. Climate Action 100+ investors currently engage with 166 companies that produce over 80 percent of the world’s global industrial emissions.^148^ By leveraging the power of capital markets, Climate Action 100+ has generated substantial commitments from some of the world’s largest polluters. For instance, as a result of engagement by a lead investor who was part of Climate Action 100+, BP now aims to reduce its operational emissions by 50 percent by 2030, up from 30-35 percent, making it the first major oil and gas provider to put in place a net-zero target covering both its upstream and downstream emissions.^149^ Investor initiatives hold immense power and opportunity for international collaborations to drive climate change mitigation and could also prove even more persuasive if they incorporated the health-related costs and expenses associated with climate change into their assessments both in terms of the cost of unabated climate change and the co-benefits to corporations of greener policies and practices.

## V. Conclusion

The future is here. The evidence is overwhelming that global warming is currently extracting a terrible price in lives and human health. As a result, governments are obliged to take aggressive climate action. While entirely justifiable on moral grounds, the economic case for rapid climate mitigation is also highly compelling, especially if the health-related costs (morbidity and mortality, health care costs, labor costs, costs of climate-related damage), as well as the substantial co-benefits to population health from mitigation are accurately accounted for and included in cost-benefit calculations underpinning climate policy.

We urge the case for international cooperation and collaboration as an underdeveloped direction for policy and action, in addition to aspirational state compliance with voluntary targets. The first green shoots are appearing, from relatively low-stakes activities like information-sharing of best practices through to exciting developments which could really “move the needle,” such as binding international trade agreements to comply with climate goals and strong financial incentives like green tariffs. Private markets can also play a part if investors take a medium to long term view on financial investments and sustainability.

Leaving the final word to the Lancet Countdown, “a future of improved health, reduced inequity, and economic and environmental sustainability … will only be possible if the world acts together to ensure that no person is left behind.” ^150^

## Note

The authors have no conflicts to disclose.
